# Supplementary material for: Bridging tumor biology and radiomics through an ultra-large physics-driven vascular model
Source: NPJ Biomed Innov. 2026 Jul 14;3:40. doi: 10.1038/s44385-026-00092-8 (PMC13369914; doi:10.1038/s44385-026-00092-8)
Supplement: Supplementary file 1 — TumorManuscript_supplementary_2026 [file 44385_2026_92_MOESM1_ESM.docx]

**Supplementary materials**

**S1 Additional Results**

**S1.1 Impact of Growth Parameters**

**Changing Angiogenic Sprouting Rate**

|  |  | Suppressed | Baseline | Enhanced |
| --- | --- | --- | --- | --- |
| Tumor Growth | **Sprouting Scaling** | 0.5 | 1.0 | 1.5 |
|  | **Growth Time [day]** | 14 | 14 | 14 |
|  | **Tumor Volume [mm^3^]** | 9.91 | 13.08 | 14.22 |
|  | **Necrosis Volume [mm^3^]** | 1.430 | 0.000 | 0.006 |
|  | **Daily Growth [%]** | 59.7 | 62.9 | 63.9 |
| Tissue Characteristics | **Cell density** | 0.868 ± 0.396 | 1.050 ± 0.090 | 1.044 ± 0.116 |
|  | **Tissue Oxygen [mmHg]** | 24.0 ± 10.9 | 27.7 ± 6.7 | 29.1 ± 5.8 |
|  | **Vas Vol Density [%]** | 1.6 ± 3.7 | 2.2 ± 4.5 | 2.4 ± 5.3 |
|  | **Tissue Perfusion [ml/g/min]** | 22.8 ± 107.0 | 24.2 ± 108.5 | 34.4 ± 142.5 |
| Vasculature Characteristics | **Perfused/Total Length Density [mm/mm^3^]** | 49.3 / 105.6 | 79.8 / 136.7 | 81.4 / 131.2 |
|  | **Perfused/Total Surface Density [mm^2^/mm^3^]** | 2.6 / 4.2 | 4.1 / 5.8 | 4.4 / 5.8 |
|  | **Perfused/Total Volume Density [%]** | 1.3 / 1.6 | 1.8 / 2.2 | 2.0 / 2.4 |
|  | **Blood Flow [nl/min]** | 15.4 ± 85.9 | 10.6 ± 59.3 | 15.7 ± 71.8 |
|  | **Blood Flow Velocity [mm/s]** | 0.58 ± 1.89 | 0.44 ± 1.34 | 0.60 ± 1.62 |
|  | **WSS [dyn/cm^2^]** | 7.0 ± 16.0 | 5.6 ± 11.7 | 7.1 ± 13.5 |
|  | **Branching Length [μm]** | 69.0 ± 76.9 | 45.5 ± 55.4 | 38.2 ± 49.2 |
|  | **Bifurcation Density [mm^-3^]** | 860 | 1797 | 2103 |

*Table S1 Summary of tumor samples with suppressed, baseline, and enhanced angiogenesis sprouting.*

**S1.2 Definition of Property Maps**

At the conclusion of each simulation, key tumor development variables are exported from COMSOL to MATLAB and interpolated from tetrahedral finite element mesh onto a Cartesian grid of isotropic voxels (50, 100, 150, or 200 microns) using MATLAB's 'griddata' function. This process prepares the data for in-depth tumor characterization and analysis. Subsequently, comprehensive property maps are generated on this grid and analyzed, covering various aspects such as tumor and necrosis segmentation, distributions of tissue oxygen partial pressure, cell density, metabolism intensity, hypoxia, proliferation activity, as well as voxel-wise distributions of tissue perfusion and blood volume fraction. By analyzing these ground truth maps—unaffected by imaging-related limitations such as signal-to-noise ratio, contrast mechanisms, or resolution constraints—we focus on identify inherently the most informative properties and optimal spatial resolutions for tumor assessment, offering insights to guide future imaging strategies and technology development.

Although the property maps generated for analysis are not meant to mimic medical images but are ground truth directly calculated from the simulation results, these properties have the potential to be non-invasively imaged in vivo. For instance, cell density might be inferred from ADC MRI, which provides information on the extracellular fluid fraction, or from CT scans that reflect atomic composition. Tissue blood perfusion and volume could be captured using various imaging modalities with contrast agents, while extracellular oxygen levels might be gauged through Electron Paramagnetic Resonance (EPR)^1^ imaging. Tissue hypoxia could be visualized using 18F-Fluoromisonidazole (FMISO) PET^2^, oxygen metabolism through Oxygen-17 MRI^3^, and proliferation rates through [18F]-FLT-PET^4^. Table S2 listed possible modalities that could potentially reflect the tumor properties of interest.

| Property Map | Imaging Modality | Clinical Resolution | Preclinical Resolution |
| --- | --- | --- | --- |
| Cell Density | CT, ADC MRI | 1-2 mm^5^ | 0.5-0.1 mm^6^ |
| Oxygen Level | EPR^1^ | Limited clinical use | 0.5-1 mm^7^ |
| Oxygen Metabolism | Oxygen-17 MRI | \|  \| \| --- \|   2-3 mm^8^ | < 2-3 mm^8^ |
| Volumetric Proliferation Rate | [18F]-FLT-PET^4^ | 4-7 mm^9^ | 1 mm^10^ |
| Hypoxia | FMISO^2^ | 4-7 mm^9^ | 1 mm^10^ |
| Tissue Perfusion | Perfusion CT, Perfusion MRI | 1-2 mm^5^ | 0.1-0.2 mm^11^ |
| Blood Volume | Perfusion CT, Perfusion MRI | 1-2 mm^5^ | 0.1-0.2 mm^11^ |

*Table S2 Imaging modalities reflecting tumor properties.*

The cell density map presents a relative estimation of solid tissue mass density, whose voxel intensity is defined as:

$$\begin{aligned} I_{\rho}=\frac{1}{J_{e}}\#s\left( 1 \right) \end{aligned}$$

The oxygen metabolism map is calculated as the relative volumetric oxygen consumption rate, aligned with the volumetric oxygen consumption rate term in the oxygenation simulation, defined as:

$$\begin{aligned} I_{OCR}=\frac{1}{J_{e}}\frac{M_{max}P_{oxy}}{P_{oxy}+P_{M50}}\#s\left( 2 \right) \end{aligned}$$

Similarly, the volumetric proliferation activity map measures the proliferation events within a voxel:

$$\begin{aligned} I_{pro}=\frac{1}{J_{e}}\frac{P_{oxy}}{P_{\lambda50}+P_{oxy}}\cdot H_{V}\#s\left( 3 \right) \end{aligned}$$

The hypoxia map in our study is designed to replicate the signal observed in 18F-Fluoromisonidazole (FMISO) PET imaging. While this map reflects tissue oxygen levels, it specifically highlights regions of low oxygenation and deliberately excludes necrotic areas from contributing to the signal. Consequently, it offers distinct insights with a particular emphasis on hypoxic yet viable tissue regions. The binding rate of FMISO as a function of oxygen partial pressure for living cells is given as^12^:

$$\begin{aligned} k_{b}\left( P_{oxy} \right)=\frac{1}{J_{e}}\frac{k_{b0}P_{50b}}{P_{oxy}+P_{50b}}\cdot H_{V}\#s\left( 4 \right) \end{aligned}$$

Where $k_{b0}$ is the maximum binding rate at $k_{b0}=4.5\times{10}^{-4} s^{-1}$, and $P_{50b}$ is the oxygen level at the half-maximum binding rate, given as 1.4 mmHg.

**S1.3 Example of Noisy Images**


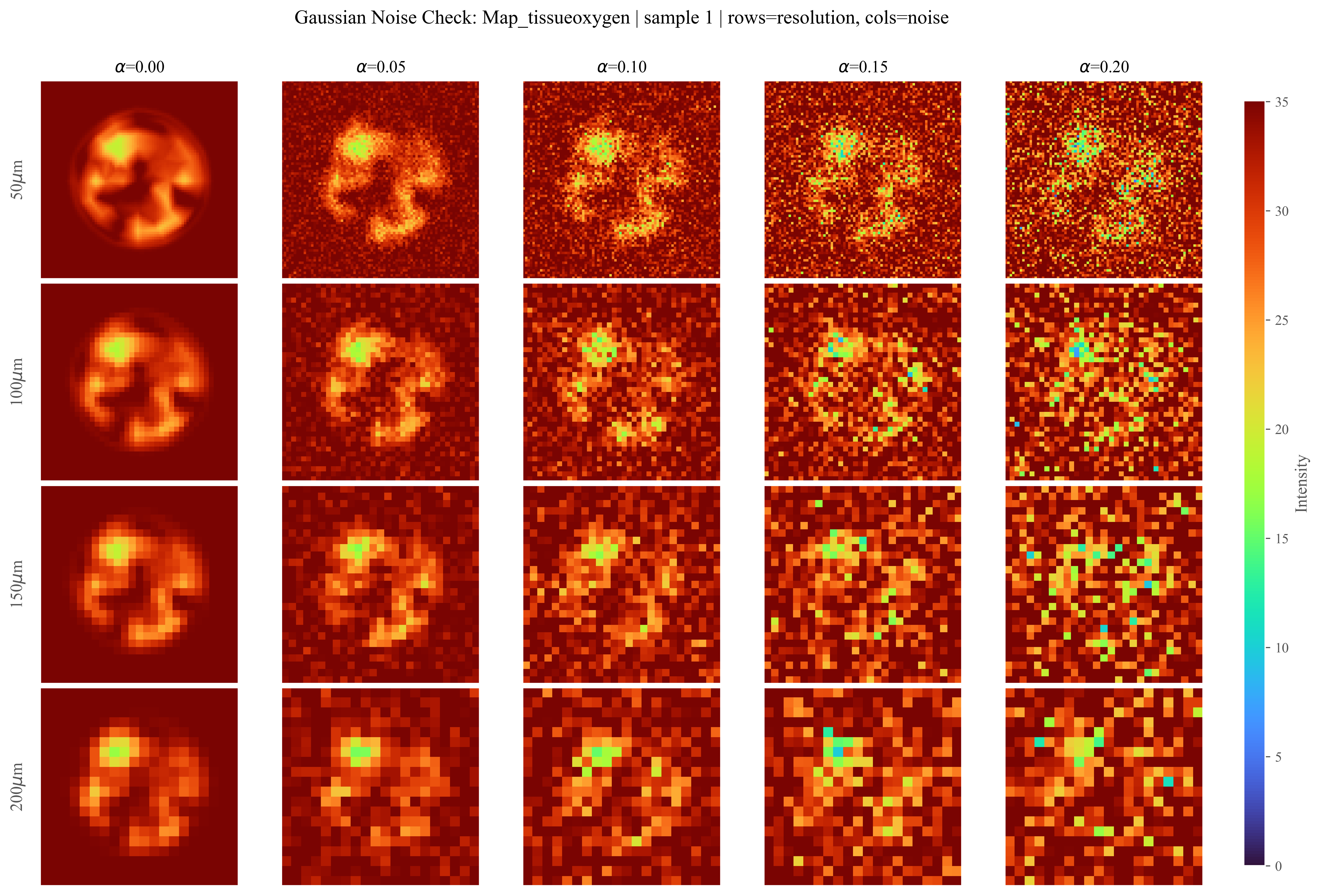


*Fig. S1 | Example of the property map with additive Gaussian noise*

Representative central slices of the simulated tissue oxygen property map are shown across spatial resolutions (rows: 50, 100, 150, and 200 µm isotropic voxel size) and noise levels (columns: α = 0.00–0.20). Additive Gaussian noise was applied with standard deviation proportional to the global mean intensity of each image (σ = α·μ), followed by non-negative clipping.

**S1.4 Biology–Radiomics Mechanistic Mapping Table**

| **Target Biological Parameter** | **Emergent Mechanistic Effect** | **Dominant Property Map** | **Representative Radiomic Feature** | **Direction of Association** | **Mechanistic Interpretation** |
| --- | --- | --- | --- | --- | --- |
| **Proliferation Rate (PR)** | Accelerated volumetric expansion outpaces angiogenesis | Relative Cell Density | First-order 90th Percentile | Increases with PR | Higher PR produces a denser viable rim surrounding hypoxic/necrotic core. |
|  | Diffusion-limited hypoxia and necrotic core formation | Hypoxia Map | NGTDM Complexity (wavelet high-pass) | Decreases with PR | Large necrotic cores generate spatially smoother hypoxia regions. |
|  | Increased structural irregularity at tumor boundary | Cell Density (wavelet filtered) | GLCM IMC2 | Decreases with PR | Increased textural irregularity reflects heterogeneous rim–core interface. |
|  | Strong radial oxygen gradients | Oxygen Map (LoG filtered) | 90th Percentile (LoG) | Increases with PR | Sharp oxygen transitions between viable rim and necrotic core. |
| **Oxygen Consumption Rate (OCR)** | Elevated metabolic demand stimulates angiogenesis | Blood Volume Map | GLSZM Small Area Low Gray-Level Emphasis (SALGLE) | Decreases with OCR | High OCR reduces avascular low-intensity regions. |
|  | Increased vessel recruitment and branching | Vascular Semantic Features | Perfused Vessel Length Fraction | Increases with OCR | Greater fraction of functional vessels supports metabolic demand. |
|  | Increased microvascular density | Blood Perfusion Map | First-order Mean | Increases with OCR | Enhanced perfusion is required to meet oxygen demand. |
|  | Reduced sharply demarcated necrotic regions (moderate PR) | Oxygen Map (LoG filtered) | 90th Percentile (LoG) | Decreases with OCR | Denser vasculature lead to less extreme oxygen gradients |

*Table S3 Mechanistic Mapping Between Tumor Biophysical Parameters and Radiomic Signatures*

**S2 Additional Methods**

**S2.1 Blood Flow Hemodynamics**

**In vivo Blood Viscosity**

Modeling blood flow within a given large vasculature poses a significant challenge. The Navier–Stokes equations for the fluid can be difficult and expensive to solve, and the presence of cellular components, which deviate the blood's behavior from the homogeneous fluid, particularly in micro-vasculatures, makes the problem even more complex.

An alternative strategy involves assuming Hagen–Poiseuille flow within each vessel segment and subsequently adjusting the apparent viscosity using empirical formulas derived from experimental data^13^. This approach has gained widespread acceptance and, to the best of our knowledge, remains the only appropriate method for addressing blood flow in large microvascular systems. Therefore, in this study, we embrace this framework for modeling the hemodynamics of vasculature.

Assuming Hagen–Poiseuille flow in the lumen of the vessel segment $e_{k}$ where $f\left( e_{k} \right)=\left\{ v_{i},v_{j} \right\}$, the blood flow rate $Q_{k}$ goes:

$$\begin{aligned} Q_{k}=G_{k}\cdot\left( P_{i}-P_{j} \right)\#s\left( 5 \right) \end{aligned}$$

$$\begin{aligned} G_{k}=\frac{\pi\cdot R_{k}^{4}}{8\cdot\mu_{k}\cdot L_{k}}\#s\left( 6 \right) \end{aligned}$$

Where $G_{k}$ is the hydraulic conductance, and $\mu_{k}$ is the apparent viscosity of the blood flowing within. $R_{k}$ and $L_{k}$ stands for the radius and the length of the vessel segment. The reference rat blood plasma's apparent viscosity $\mu_{0}$ measured at 37$℃$ is 1.05 cP^14^. An additional two-step correction is required to account for various vessel diameters and red blood cell (RBC) concentration conditions and estimate realistic in vivo apparent viscosity in blood vessels.

The first correction is for in vitro viscosity that considers the plasma cell-free layer due to the centering movement of erythrocytes resulting in lower resistance between flow and tube wall, also known as the Fahraeus-Lindqvist Effect^15^. A. R. Pries et al.^16^ proposed an empirical correction for such an effect based on in vitro glass tube experiments:

$$\begin{aligned} \eta_{vitro}=1+\left( \eta_{0.45}-1 \right)\cdot\frac{\left( 1-H_{d} \right)^{C}-1}{\left( 1-0.45 \right)^{C}-1}\#s\left( 7 \right) \end{aligned}$$

$$\begin{aligned} \eta_{0.45}=220\cdot e^{-1.3D}+3.2-2.44\cdot e^{-0.06D^{0.645}}\#s\left( 8 \right) \end{aligned}$$

$$\begin{aligned} C=\left( 0.8+e^{-0.075D} \right)\cdot\left( \frac{1}{1+{10}^{-11}\cdot D^{12}}-1 \right)+\frac{1}{1+{10}^{-11}\cdot D^{12}}\#s\left( 9 \right) \end{aligned}$$

Where $D$ is the measured anatomic vessel diameter in microns, $\eta_{vitro}$ is the relative apparent viscosity and $\eta_{0.45}$ is that of rat blood with discharge hematocrit at a level of 0.45. The term discharge hematocrit is defined as the volume fraction of RBCs delivered by the blood flow^17^. The second correction accounts for the endothelial layer (ECL) that observed to substantially increase the in vivo blood viscosity^18^. The effective thickness of the layer $W_{eff}$ can be calculated by adding an asymptotic component $W_{as}$ and a biphasic component $W_{peak}$ with a peak^14^:

$$\begin{aligned} W_{eff}=W_{as}+W_{peak}\cdot\left( 1+{1.18\cdot H}_{d} \right)\#s\left( 10 \right) \end{aligned}$$

$$\begin{aligned} W_{as}=\left\{ \begin{aligned} 0 \#if D\leq2.4 \\ 2.6\cdot\frac{D-2.4}{D+100-4.8} \#if D>2.4 \end{aligned} \right.\#s\left( 11 \right) \end{aligned}$$

$$\begin{aligned} W_{peak}=\left\{ \begin{aligned} 0 \#if D\leq2.4 \\ 1.1\cdot\frac{D-2.4}{10.5-2.4} \#if 2.4<D\leq10.5 \\ 1.1\cdot e^{-0.03\cdot\left( D-10.5 \right)} \#if 10.5<D \end{aligned} \right.\#s\left( 12 \right) \end{aligned}$$

Then the effective diameter and in vivo relative apparent blood viscosity $D_{eff}$ reads:

$$\begin{aligned} D_{eff}=D-2W_{eff}\#s\left( 13 \right) \end{aligned}$$

$$\begin{aligned} \eta_{vivo}=\eta_{vitro}\cdot\left( \frac{D}{D_{eff}} \right)^{4}\#s\left( 14 \right) \end{aligned}$$

**Linear System Construction for Blood Flow**

With the in vivo viscosity and boundary conditions given, a linear system can be constructed by applying mass conservation to all the vertices in the vasculature graph.

$$\begin{aligned} G^{M}\cdot P^{M}=Q^{M}\#s\left( 15 \right) \end{aligned}$$

Where $G^{M}$ is a sparse symmetric matrix for hydraulic conductance of all vessel segments with

$$\begin{aligned} G_{ij}^{M}=\left\{ \begin{aligned} -G_{i,j} &i\neq j \\ \sum_{v_{k}\in V} G_{i,k} &i=j \end{aligned} \right.\#s\left( 16 \right) \end{aligned}$$

$$\begin{aligned} with G_{i,j}=\sum_{e_{k}\in E_{i,j}} G_{k}\#s\left( 17 \right) \end{aligned}$$

Where $E_{i,j}$ is the set contains all the edges that connect both vertices $v_{i}$ and $v_{j}$. $P^{M}$ is the vector of blood pressure at vertices, and $Q^{M}$ is the vector of the blood flow rate of net outflow from vertices. By splitting inner vertex and boundary vertex-related terms into different sides of the equation, the linear system can be transformed to^19^:

$$\begin{aligned} G_{int}^{M}\cdot P_{int}^{M}=G_{b}^{M}\cdot P_{b}^{M}\#s\left( 18 \right) \end{aligned}$$

$G_{int}^{M}$ is the submatrix of the hydraulic conductance matrix that contains inner vertex rows, while $G_{b}^{M}$ only contains boundary vertex rows. With given boundary blood pressure values $P_{b}^{M}$, the unknown inner vertex blood pressure $P_{int}^{M}$ can be effectively solved using the generalized minimum residual method (GMRES)^20^

**Phase Separation Effect**

Red blood cells (RBC) have a highly heterogeneous distribution in real vasculature, especially in poorly structured tumor vasculatures^21^, due to the nonproportional distribution of RBCs in daughter branches at diverging bifurcations known as the phase separation effect^22^. It is important to obtain an estimation of RBC distribution in vasculature not only because of its effect on apparent blood viscosity but also because of its dominating role in oxygen delivery. Approximately 98% of the oxygen carried in the blood is bound to hemoglobin contained in RBCs, while only 2% is dissolved in plasma and RBC water^23^. Vessels without adequate red blood cells are unable to support the tissue metabolism despite the flow rate.

In this study, we assume a uniform discharge hematocrit feed of 0.45^24^ at the vasculature inlet. In the downstream bifurcation vertices in the vasculature, we adopted an experimentally determined parametric description of the phase separation effect by Pries AR et al.^14^:

$$\begin{aligned} logit\left( {FQ}_{E} \right)=A+B\cdot logit\left( \frac{{FQ}_{B}-X_{0}}{1-2X_{0}} \right)\#s\left( 19 \right) \end{aligned}$$

$$\begin{aligned} A=-13.29\cdot\frac{\frac{{D_{\alpha}}^{2}}{{D_{\beta}}^{2}}-1}{\frac{{D_{\alpha}}^{2}}{{D_{\beta}}^{2}}+1}\cdot\frac{1-H_{d}}{D_{p}}\#s\left( 20 \right) \end{aligned}$$

$$\begin{aligned} B=1+6.98\cdot\frac{1-H_{d}}{D_{p}}\#s\left( 21 \right) \end{aligned}$$

$$\begin{aligned} X_{0}=0.964\cdot\frac{1-H_{d}}{D_{p}}\#s\left( 22 \right) \end{aligned}$$

$D_{p}$, $D_{\alpha}$ and $D_{\beta}$ are the vessel diameters of the parent vessel and two daughter vessels measured in microns. $H_{d}$ is the discharge hematocrit (Hd) of the parent vessel. ${FQ}_{E}$ is defined as the fractional flow of RBCs into the daughter branch $\alpha$ and ${FQ}_{B}$ is the corresponding blood flow fraction. The RBC distribution at each bifurcation-vertex can be calculated as shown above using a bisection method. Combining this with a depth-first search (DFS)-like algorithm, the $Hd$ distribution in the entire vasculature network can be obtained efficiently.

**Iterative Update**

Discharge hematocrit levels significantly influence the apparent viscosity of blood in each vessel segment, potentially altering the flow patterns within the vasculature system. To tackle the interaction between blood flow and discharge hematocrit distribution, we employ an iterative approach. This involves solving a linear system for blood flow and conducting vasculature traversal for discharge hematocrit until the system reaches convergence. Typically, with a physiologically plausible vasculature structure, convergence is achieved with low iteration differences within 3-4 iterations.

**Edge Contraction**

The node-tube representation of vasculature data effectively captures vascular morphology and vessel shape. However, the extensive number of nodes and tubes could significantly complicate the computation of blood flow. To improve computational efficiency, we developed a corresponding vertex-edge model specifically for hemodynamic calculations within the vasculature. This approach leverages the fact that non-leaking edges exhibit consistent flow through all constituent tubes, meaning that only the aggregate information of each edge and the pressure at its vertices are required to assess perfusion. By applying edge contraction to the vasculature, conducting hemodynamic computations on this equivalent vertex-edge model, and then mapping the results back to the original node-tube representation, we significantly enhance computational efficiency, achieving improvements by orders of magnitude.

**S2.2 Initialization and boundary conditions**

**Oxygen Dynamics in Host Tissue**

Despite the high degree of microvascular heterogeneity arising from both extrinsic and intrinsic factors, control mechanisms in healthy tissue effectively mitigate variations in oxygen supply, resulting in a relatively stable and adequate oxygen concentration^25^. This observation is supported by studies such as that by Carreau et al.^26^, which report low spatial variation in oxygen concentration in various healthy organs, including the brain, muscle, and intestinal tissue. In light of these findings, and to avoid the need to explicitly model the healthy tissue vasculature, we proposed a dynamic equilibrium oxygen partial pressure distribution in healthy tissue governed by:

$$\begin{aligned} \frac{\partial P_{oxy}}{\partial t}=\nabla\cdot\left( D_{oxy}\nabla P_{oxy} \right)-\frac{1}{a_{oxy}}M\left( P_{oxy} \right)+\frac{1}{a_{oxy}}S_{h}\left( P_{boxy}-P_{oxy} \right)\#s\left( 23 \right) \end{aligned}$$

$S_{h}$ represents the oxygen supply from healthy tissue vasculature, the value is selected such that the steady state oxygen concentration in normal tissue equals $P_{hs}$ :

$$\begin{aligned} M\left( P_{hs} \right)=S_{h}\left( P_{boxy}-P_{hs} \right)\#s\left( 24 \right) \end{aligned}$$

**Host Vasculature Initialization**

The vascularization through angiogenesis requires pre-existing host vasculature to start with. Common methods establishing host vasculature includes the cubic grid vasculature^27^, parallel vessels arrays^28^, and reduced vasculature data containing a few major vessels^29^. However, existing methods can hardly fulfill our need for unbiased tumor growth. The artificial vessel arranged as cubic grid or paralleled lines could introduce bias in certain growth direction while the density of reduced vasculature could be inadequate for nutrition supply and angiogenesis initiation. Ideally, the entire vasculature extracted from normal tissue is preferred for host vasculature initialization, however, with the scarcity of data availability and the cost of handing a much larger simulation region and vasculature size, makes it currently impractical. As an alternative approach, we propose a novel spatially stratified tangent vessel method for host vasculature initialization. This method provides a simple and theoretically unbiased vascular environment for tumor development and allows for random sampling to further annihilation the risk of directional growth bias introduced error in statistical analysis.

In our model, the initial tumor assumes a spherical shape with a radius $R_{Tumor}^{init}=150 \mu m$. The environmental vasculature surrounding the tumor is organized as tangent lines on an extended sphere situated $L_{S2V}^{init}=50 \mu m$ away from the tumor’s surface. Both the tangent points and vessel orientations on the tangent plane are randomly sampled. To mitigate the risk of large vessel-starved areas, which are not physiologically reasonable in a healthy host, we implement a stratification strategy. This involves sampling the tangent points part by part within uniformly divided subregions on the surface of the sphere. Each initialized vessel is 6 μm in radius, with a total length of $L_{HostVes}^{init}=1000 \mu m$.

**Boundary Blood Pressure**

The boundary blood pressure assignment is also crucial for proper perfusion and the downstream vessel remodeling estimation. For a static system, convex optimization method considering the WSS under a mass conservation constraint^13^ could be used to estimate the boundary blood pressure. However, the optimization-based estimation is impractically expensive for an evolving vasculature. Tailored to our dynamic development task and the specific host vasculature morphology, we introduce a novel location-encoded blood pressure assignment method to determine the inlet and outlet blood pressures of the vasculature under significant deformation. This method incorporates two key components.

**Distance-****to-****center-based baseline pressure term**: This term adjusts the blood pressure based on the distance from the vessel center. This ensures a progressive increase in pressure difference between the inlet and outlet as the vessel length extends.

**Angular-position-based pressure variation term**: This term introduces a blood pressure shift for host vessels with different orientation and facilitates the establishment of appropriate pressure gradients within neo-vasculatures connecting different host vessels, especially in the central region.

For the location-encoded blood pressure assignment method, the initial step involves estimating the vessel length based on the position of the inlet or outlet. We assume that throughout the near-spheroidal tumor growth, for the host vessel ends, their distance to tumor surface and angular position with respect to tumor center remains constant. And for other parts in host vasculature, their distance to tumor surface does not drop below initial surface to vessel distance $L_{S2V}^{init}$. Based on these assumptions, we introduce another geometric approximation to decompose any host vessel into three components: one circular arc component on the extended sphere surface, and two tangent line components connecting the vessel ends and the arc.

The constant host vessel to tumor surface distance and the constant angle between two ends of a host vessel with respect to the tumor center throughout the tumor development is:

$$\begin{aligned} L_{S2E}=\sqrt{\left( R_{Tumor}^{init}+L_{S2V}^{init} \right)^{2}+\left( \frac{L_{HostVes}^{init}}{2} \right)^{2}}-R_{Tumor}^{init}\#s\left( 25 \right) \end{aligned}$$

$\begin{aligned} \theta_{ECE}=2arctan\left( \frac{L_{HostVes}^{init}}{2\left( R_{Tumor}^{init}+L_{S2V}^{init} \right)} \right)\#s\left( 26 \right) \end{aligned}$as the tumor grows, the estimated distance from tumor center to the arc sphere at time t is:

$$\begin{aligned} L_{C2V}^{t}=L_{C2E}^{t}-L_{S2E}+L_{S2V}^{init}\#s\left( 27 \right) \end{aligned}$$

Where $L_{C2E}^{t}$ represents the distance from the evaluated vessel end to the tumor center at time t, which is the only required variable. The angle between vessel end and the corresponding tangent point is:

$$\begin{aligned} \theta_{ECT}^{t}=\arccos\left( \frac{L_{C2V}^{t}}{L_{C2E}^{t}} \right)\#s\left( 28 \right) \end{aligned}$$

And the total length of the deformed host vessel is written as:

$$\begin{aligned} L_{vas}^{t}=2L_{C2E}^{t}\left( \theta_{ECE}-\theta_{ECT}^{t}+\sin\left( \theta_{ECT}^{t} \right) \right)\#s\left( 29 \right) \end{aligned}$$

Finally, the baseline pressure term is:

$$\begin{aligned} P_{D2C}^{t}\left( L_{vas}^{t} \right)=\pm\kappa_{D2C}\frac{L_{vas}^{t}}{2}+P_{ref}\#s\left( 30 \right) \end{aligned}$$

Where $P_{ref}$ is the reference mean blood pressure of the vasculature, $\kappa_{D2C}$ is the gradient for distance-based blood pressure term, which is determined according to the reference WSS and radius of the vasculature. Sign in the first term determines the flow direction which is randomly assigned during initialization with a positive gradient for inlet and a negative for outlet.

The angular position of the vessel end can be calculated as:

$$\begin{aligned} \theta_{C2E}^{t}=\arccos\left( {\hat{\boldsymbol{n}}}_{C2E}^{t}\cdot\hat{\boldsymbol{z}} \right)\#s\left( 31 \right) \end{aligned}$$

$\begin{aligned} \varphi_{C2E}^{t}=arctan\left( \frac{{\hat{\boldsymbol{n}}}_{C2E}^{t}\cdot\hat{\boldsymbol{y}}}{{\hat{\boldsymbol{n}}}_{C2E}^{t}\cdot\hat{\boldsymbol{x}}} \right)\#s\left( 32 \right) \end{aligned}$ ${\hat{\boldsymbol{n}}}_{C2E}^{t}$ is the unit vector pointing from tumor center to the vessel end, θ is the polar angle and φ is the azimuthal angle. $\hat{\boldsymbol{x}}$, $\hat{\boldsymbol{y}}$, and $\hat{\boldsymbol{z}}$ are unit axis vectors of the cartesian coordinate system.

The angular-position-based pressure term is:

$$\begin{aligned} P_{\mathrm{angle}}^{t}= c_{angle}\kappa_{D2C}\frac{L_{C2E}^{t}}{\sqrt{2}}\left[ \cos\left( 2\theta_{C2E}^{t} \right)+\sin\left( 2\theta_{C2E}^{t} \right)\sin\left( 2\varphi_{C2E}^{t} \right) \right]\#s\left( 33 \right) \end{aligned}$$

Where $c_{angle}$ represents the relative strength of angular variation compared to the baseline gradient term. This low-frequency angular term ensures that vessel ends positioned at approximately opposite angles share similar pressure variation values. This guarantees approximately constant blood pressure gradients for all host vessels throughout their growth.

**S2.3 Additional Vasculature Remodeling**

**Tissue-induced motion**

In our model, the vasculature is embedded within deforming tissue and adapts its shape and length accordingly. The movement speed of a vasculature vertex located at position $\boldsymbol{x}$ with the deformation of tissue can be written as

$$\begin{aligned} \boldsymbol{v}\left( \boldsymbol{x},t \right)=\boldsymbol{V}\left( \phi^{-1}\left( \boldsymbol{x},t \right),t \right)\boldsymbol{\#}s\left( 34 \right) \end{aligned}$$

Where $\phi$ is the deformation function mapping the material space to world space, and $\phi^{-1}$ is its inverse. $\boldsymbol{V}$ denotes the velocity of the tissue as defined in material space. Due to the substantial tissue deformation caused by tumor growth, some vessel segments may be stretched to long lengths and lose the ability to capture vessel tortuosity. To maintain modeling accuracy, we insert extra vertices to ensure that the length of each vessel segment remains approximately 20 microns.

**Angular Remodeling**

In addition to tissue-induced motion, we account for the potential of vessels to move through tissue due to longitudinal tension^30^ ^31^. This mechanism helps to align the branching angles closer to 120 degrees, as observed in microvascular networks^32^. The resultant ‘force’ of vessel tensions acting at each network vertex can be modeled as^33^:

$$\begin{aligned} \mathbf{f}_{t}=\frac{\left( \sum D_{i}\boldsymbol{n}_{i} \right)\left( \sum D_{i} \right)}{\sum D_{i}L_{i}}\#s\left( 35 \right) \end{aligned}$$

$i$ indexes the vessel segments connecting to the examined vertex, $D$ and $L$ represent the corresponding vessel diameter and length. $\boldsymbol{n}$ is the unit vector indicating vessel orientation, taking the examined vertex as the starting point. They will migrate if the net force exceeds a threshold $F_{th}$:

$$\begin{aligned} \boldsymbol{v}=v_{bamax}\mathbf{f}_{t}\left( 1-\frac{F_{bath}}{\left\| \mathbf{f}_{t} \right\|_{2}} \right)\boldsymbol{\#}s\left( 36 \right) \end{aligned}$$

Where $v_{bamax}$ is the maximum velocity, keeping this velocity relatively low is very important for the numerical stability of branching angle remodeling. The threshold $F_{bath}$ is included to prevent the vasculature from losing its curved structures^32^.

**S2.4 Model Parameters**

**Continuum model parameters**

| Parameter | Description | Value | Unit | Reference |
| --- | --- | --- | --- | --- |
| $\boldsymbol{\phi}_{\boldsymbol{Host}}$ | Blatz-Ko model parameter | 1 |  | Richard Moran et al.^34^ |
| $\boldsymbol{\beta}_{\boldsymbol{Host}}$ | Blatz-Ko model parameter | 2 |  | Richard Moran et al.^34^ |
| $\boldsymbol{\mu}_{\boldsymbol{Host}}$ | Host shear modulus | 1 | $kPa$ | Richard Moran et al.^34^ |
| $\boldsymbol{\phi}_{\boldsymbol{Tumor}}$ | Blatz-Ko model parameter | 0.2 |  | James MacLaurin^35^ |
| $\boldsymbol{\beta}_{\boldsymbol{Tumor}}$ | Blatz-Ko model parameter | 4 |  | James MacLaurin^35^ |
| $\boldsymbol{\mu}_{\boldsymbol{Tumor}}$ | Tumor shear modulus | 2.7 | $kPa$ | James MacLaurin^35^ |
| $\boldsymbol{P}_{\boldsymbol{N}}$ | Critical $P_{oxy}$ for necrosis | 0.1 | $mmHg$ |  |
| $\boldsymbol{\rho}$ | Tissue density | 1 | $\frac{g}{ml}$ |  |
| $\boldsymbol{K}_{\boldsymbol{g}}$ | Growth rate | 0.693 | ${day}^{-1}$ | See text |
| $\boldsymbol{P}_{\boldsymbol{\lambda}\boldsymbol{50}}$ | $P_{oxy}$ at half maximum growth rate | 10 | $mmHg$ | See text |

*Tabel S4 Biophysical parameters used in the tumor growth model.*

**Discrete model parameters**

| Module | Parameter | Description | Value | Unit | Reference |
| --- | --- | --- | --- | --- | --- |
| Oxygen | $D_{oxy}$ | Oxygen diffusion coefficient | 2410 | ${\mu m}^{2}s^{-1}$ | Bentley et al.^36^ |
|  | $a_{oxy}$ | Oxygen solubility | 38.9 | $nlO_{2}{ml}^{-1}{mmHg}^{-1}$ | Bentley et al.^36^ |
|  | $\rho$ | Tissue mass density | 1000 | $kg\cdot m^{-3}$ |  |
|  | $M_{max}$ | Max oxygen consumption | 2-4 | $mmHg{\cdot s}^{-1}$ | See text |
|  | $P_{M50}$ | $P_{oxy}$ with half -maximum consumption | 1 | $mmHg$ | Goldman^37^ |
|  | $P_{boxy}$ | Blood $P_{oxy}$ | 35.5 | $mmHg$ |  |
|  | $P_{hs}$ | Steady state host tissue $P_{oxy}$ | 35 | $mmHg$ | Carreau et al.^26^ |
|  | $P_{cap}$ | Max oxygen pressure difference across vessel wall | 1 | $mmHg$ |  |
| Perfusion | $\mu_{0}$ | Apparent viscosity of rat blood at 37$℃$ | 1.05 | $mPa\cdot s$ | Pries et al.^14^ |
|  | $P_{ref}$ | Reference mean blood pressure | 40 | $mmHg$ |  |
|  | $\kappa_{D2C}$ | Gradient for distance-based blood pressure term | 1.8752 | $mmHg{\cdot mm}^{-1}$ |  |
|  | $c_{angle}$ | Relative strength of angular blood pressure variation | 2 |  |  |
| TAF | $C_{max}$ | Max TAF concentration | 1 |  |  |
|  | $D_{TAF}$ | TAF Diffusion coefficient | 20 | ${\mu m}^{2}s^{-1}$ | Alberding et al.^33^ |
|  | $K_{TAF}$ | TAF decay rate | 0.002 | $s^{-1}$ | Adapted from Alberding et al.^33^ |
|  | $P_{TAF}$ | Tissue $P_{oxy}$ where cells start to release TAF | 34.5 | $mmHg$ |  |
| Angiogenesis | $k_{sprout}$ | Maximum sprout rate per length | 0.05 | ${\mu m}^{-1}{day}^{-1}$ |  |
|  | $C_{TAF50}$ | $C_{TAF}$ for half-maximal sprout rate | 0.33 |  |  |
|  | $C_{th}$ | $C_{TAF}$ threshold for sprout formation | 0.01 |  |  |
|  | $C_{mig}$ | $C_{TAF}$ threshold for stalk cell proliferation | 0.01 |  |  |
|  | $R_{sprout}$ | Radius of sprout | 6 | $\mu m$ |  |
|  | $V_{sprout}$ | Velocity of sprout elongation | 75 | $\mu m\cdot{day}^{-1}$ | Levi B. Wood et al.^38^ |
|  | $k_{TAF}$ | Weight for TAF gradient | 1 |  |  |
|  | $k_{ana}$ | Weight for anastomosis bias | 1 |  |  |
|  | $k_{rand}$ | Weight for random variation | 0.5 |  |  |
|  | $D_{ana}$ | Maximum tip cell sensing distance | 75 | $\mu m$ | Gerhardt ^39^ |
|  | $\theta_{ana}$ | Maximum vessel sensing angle | $\pi/3$ |  | Secomb et al.^32^ |
|  | $L_{ana}$ | Anastomosis threshold | 25 | $\mu m$ |  |
| Remodeling | $v_{bamax}$ | Branching angle remodeling velocity | 1 | $\mu m\cdot h^{-1}$ |  |
|  | $F_{bath}$ | Branching angle remodeling threshold | 0.25 |  | JP Alberding et al.^33^ |
|  | $T_{s}$ | Structural adaptation coefficient | 192 | $day$ |  |
|  | $n_{r}$ | Adaptation weighting factor for reference radius | 4 |  |  |
|  | $R_{ref}$ | Reference radius in adaptation | 12 | $\mu m$ |  |
|  | $\tau_{ref}$ | Reference wall shear stress | 15 | $dyn\cdot{cm}^{-2}$ | Secomb et al.^13^ |
| Initialization | $R_{Tumor}^{init}$ | Initial tumor radius | 150 | $\mu m$ |  |
|  | $L_{S2V}^{init}$ | Initial tumor surface to host vasculature distance | 50 | $\mu m$ |  |
|  | $L_{HostVes}^{init}$ | Initial host vasculature length | 1000 | $\mu m$ |  |
|  | $N_{HostVes}^{init}$ | Initial host vessel number | 50 |  |  |
|  | $L_{Host}^{init}$ | Initial host tissue cube length | 4000 | $\mu m$ |  |

*Table S5 Biophysical Parameters related to vasculature and oxygenation.*

**Reference**

1. Epel, B., Redler, G. & Halpern, H. J. How in vivo EPR Measures and Images Oxygen. *Adv. Exp. Med. Biol.* **812**, 113–119 (2014).

2. Warren, D. R. & Partridge, M. The role of necrosis, acute hypoxia and chronic hypoxia in 18F-FMISO PET image contrast: a computational modelling study. *Phys. Med. Biol.* **61**, 8596 (2016).

3. Paech, D. *et al.* Quantitative Dynamic Oxygen 17 MRI at 7.0 T for the Cerebral Oxygen Metabolism in Glioma. *Radiology* **295**, 181–189 (2020).

4. McKinley, E. T. *et al.* Limits of [18F]-FLT PET as a Biomarker of Proliferation in Oncology. *PLOS ONE* **8**, e58938 (2013).

5. Padhani, A. R. *et al.* Diffusion-Weighted Magnetic Resonance Imaging as a Cancer Biomarker: Consensus and Recommendations. *Neoplasia* **11**, 102–125 (2009).

6. Clark, D. P. & Badea, C. T. Advances in Micro-CT Imaging of Small Animals. *Phys. Medica PM Int. J. Devoted Appl. Phys. Med. Biol. Off. J. Ital. Assoc. Biomed. Phys. AIFB* **88**, 175–192 (2021).

7. Elas, M. *et al.* Quantitative tumor oxymetric images from 4D electron paramagnetic resonance imaging (EPRI): Methodology and comparison with blood oxygen level-dependent (BOLD) MRI. *Magn. Reson. Med.* **49**, 682–691 (2003).

8. Zhu, X.-H. & Chen, W. In vivo oxygen-17 NMR for imaging brain oxygen metabolism at high field. *Prog. Nucl. Magn. Reson. Spectrosc.* **59**, 319–335 (2011).

9. Rahmim, A. & Zaidi, H. PET versus SPECT: strengths, limitations and challenges. *Nucl. Med. Commun.* **29**, 193 (2008).

10. Cherry, S. R. In vivo molecular and genomic imaging: new challenges for imaging physics. *Phys. Med. Biol.* **49**, R13 (2004).

11. Wehrl, H. F. *et al.* Preclinical and Translational PET/MR Imaging. *J. Nucl. Med.* **55**, 11S-18S (2014).

12. Muz, B., de la Puente, P., Azab, F. & Azab, A. K. The role of hypoxia in cancer progression, angiogenesis, metastasis, and resistance to therapy. *Hypoxia* **3**, 83–92 (2015).

13. Fry, B. C., Lee, J., Smith, N. P. & Secomb, T. W. Estimation of Blood Flow Rates in Large Microvascular Networks. *Microcirculation* **19**, 530–538 (2012).

14. Pries, A. R. & Secomb, T. W. Microvascular blood viscosity in vivo and the endothelial surface layer. *Am. J. Physiol.-Heart Circ. Physiol.* **289**, H2657–H2664 (2005).

15. Fåhræus, R. & Lindqvist, T. THE VISCOSITY OF THE BLOOD IN NARROW CAPILLARY TUBES. *Am. J. Physiol.-Leg. Content* **96**, 562–568 (1931).

16. Pries, A. R., Neuhaus, D. & Gaehtgens, P. Blood viscosity in tube flow: dependence on diameter and hematocrit. *Am. J. Physiol.-Heart Circ. Physiol.* **263**, H1770–H1778 (1992).

17. Botkin, N. D., Kovtanyuk, A. E., Turova, V. L., Sidorenko, I. N. & Lampe, R. Accounting for Tube Hematocrit in Modeling of Blood Flow in Cerebral Capillary Networks. *Comput. Math. Methods Med.* **2019**, 4235937 (2019).

18. Pries, A. R. *et al.* Microvascular blood flow resistance: role of endothelial surface layer. *Am. J. Physiol.-Heart Circ. Physiol.* **273**, H2272–H2279 (1997).

19. Stamatelos, S. K., Kim, E., Pathak, A. P. & Popel, A. S. A bioimage informatics based reconstruction of breast tumor microvasculature with computational blood flow predictions. *Microvasc. Res.* **91**, 8–21 (2014).

20. Saad, Y. & Schultz, M. H. GMRES: A Generalized Minimal Residual Algorithm for Solving Nonsymmetric Linear Systems. *SIAM J. Sci. Stat. Comput.* **7**, 856–869 (1986).

21. Du, J. & Sheng, K. Modeling Tumor Vasculature for Perfusion and Oxygenation Analysis. in (AAPM, 2023).

22. Pries, A. R., Ley, K., Claassen, M. & Gaehtgens, P. Red cell distribution at microvascular bifurcations. *Microvasc. Res.* **38**, 81–101 (1989).

23. Pittman, R. N. Tissue Gas Transport. in *Regulation of Tissue Oxygenation* (Morgan & Claypool Life Sciences, 2011).

24. Everds, N. CHAPTER 17 - Hematology of the Mouse. in *The Laboratory Mouse* (eds Hedrich, H. J. & Bullock, G.) 271–286 (Academic Press, London, 2004). doi:10.1016/B978-012336425-8/50070-4.

25. Roy, T. K. & Secomb, T. W. Functional implications of microvascular heterogeneity for oxygen uptake and utilization. *Physiol. Rep.* **10**, e15303 (2022).

26. Carreau, A., Hafny-Rahbi, B. E., Matejuk, A., Grillon, C. & Kieda, C. Why is the partial oxygen pressure of human tissues a crucial parameter? Small molecules and hypoxia. *J. Cell. Mol. Med.* **15**, 1239–1253 (2011).

27. Shirinifard, A. *et al.* 3D Multi-Cell Simulation of Tumor Growth and Angiogenesis. *PLoS ONE* **4**, (2009).

28. Vavourakis, V. *et al.* A Validated Multiscale In-Silico Model for Mechano-sensitive Tumour Angiogenesis and Growth. *PLOS Comput. Biol.* **13**, e1005259 (2017).

29. Duswald, T., Lima, E. A. B. F., Oden, J. T. & Wohlmuth, B. Bridging scales: A hybrid model to simulate vascular tumor growth and treatment response. *Comput. Methods Appl. Mech. Eng.* **418**, 116566 (2024).

30. Gaehtgens, P. A. L. Radial and longitudinal distensibility of arterial microvessels in the mesentery and their dependence on extravascular structures. *Pflüg. Arch.* **330**, 277–289 (1971).

31. Jackson, Z. S., Gotlieb, A. I. & Langille, B. L. Wall Tissue Remodeling Regulates Longitudinal Tension in Arteries. *Circ. Res.* **90**, 918–925 (2002).

32. Secomb, T. W., Alberding, J. P., Hsu, R., Dewhirst, M. W. & Pries, A. R. Angiogenesis: An Adaptive Dynamic Biological Patterning Problem. *PLOS Comput. Biol.* **9**, e1002983 (2013).

33. Alberding, J. P. & Secomb, T. W. Simulation of angiogenesis in three dimensions: Application to cerebral cortex. *PLOS Comput. Biol.* **17**, e1009164 (2021).

34. Moran, R., Smith, J. H. & García, J. J. Fitted hyperelastic parameters for Human brain tissue from reported tension, compression, and shear tests. *J. Biomech.* **47**, 3762–3766 (2014).

35. MacLaurin, J. The buckling of capillaries in tumours. (University of Oxford, 2011).

36. Bentley, T. B., Meng, H. & Pittman, R. N. Temperature dependence of oxygen diffusion and consumption in mammalian striated muscle. *Am. J. Physiol.-Heart Circ. Physiol.* **264**, H1825–H1830 (1993).

37. Goldman, D. Theoretical Models of Microvascular Oxygen Transport to Tissue. *Microcirc. N. Y. N 1994* **15**, 795–811 (2008).

38. Wood, L. B., Ge, R., Kamm, R. D. & Asada, H. H. Nascent vessel elongation rate is inversely related to diameter in in vitro angiogenesis. *Integr. Biol.* **4**, 1081–1089 (2012).

39. Gerhardt, H. *et al.* VEGF guides angiogenic sprouting utilizing endothelial tip cell filopodia. *J. Cell Biol.* **161**, 1163–1177 (2003).
